# Acute Paraplegia in the Emergency Department: Acute Leriche Syndrome as a Vascular Mimic of a Spinal Cord Emergency

**DOI:** 10.7759/cureus.114741

**Published:** 2026-08-18

**Authors:** Rico W Putra, Dwiwardoyo Triyuliarto, Fatah Abdul Yasir, Yuli Prastio

**Affiliations:** 1 Emergency Medicine, Faculty of Medicine, Universitas Brawijaya, Malang, IDN; 2 Emergency Medicine, General Hospital Saiful Anwar, Malang, IDN

**Keywords:** acute paraplegia, aortoiliac occlusion, emergency medicine, leriche syndrome, spinal cord ischemia

## Abstract

Acute paraplegia is a neurological emergency requiring prompt diagnosis to prevent irreversible neurological deficits and permanent disability. Although traumatic causes of acute paraplegia are well-characterized, non-traumatic acute paraplegia poses a diagnostic challenge because of its diverse etiologies. Leriche syndrome, also known as aortoiliac occlusive disease, typically presents with chronic lower-extremity claudication, diminished femoral pulses, and erectile dysfunction; however, it can rarely present with acute paraplegia and mimic a spinal cord emergency. We report the case of a 58-year-old woman with uncontrolled diabetes who presented to the emergency department with sudden-onset, non-traumatic flaccid paraplegia, accompanied by sensory loss below the level of L1 and urinary retention. Physical examination revealed cold bilateral lower extremities with absent bilateral femoral and distal pulses, raising suspicion for an acute vascular etiology. Computed tomography angiography revealed complete occlusion of the abdominal aorta and bilateral common iliac arteries at the L1-L2 level, consistent with acute Leriche syndrome. The patient was treated with intravenous heparin, followed by catheter-directed thrombolysis and aortic stent placement. Her motor function improved from 0/5 at admission to 3/5 at hospital discharge, with substantial neurological recovery during the three-month follow-up while undergoing physiotherapy. This case demonstrates that acute Leriche syndrome may present with acute paraplegia and mimic a spinal cord emergency. It highlights the critical importance of incorporating vascular assessment into the evaluation of non-traumatic acute paraplegia, as timely diagnosis and revascularization may improve neurological outcomes.

## Introduction

Acute paraplegia is a neurological emergency characterized by the rapid onset of symmetrical motor and sensory loss in both lower extremities, reaching maximum severity within 21 days of symptom onset. This condition may result from a variety of underlying pathologies, including disorders of the spinal cord (myelopathy), spinal nerve roots, peripheral nerves, or muscle disease (myopathy). Early and accurate diagnosis is critical, as delayed intervention can lead to irreversible neurological deficits and long-term disability [1,2].

While traumatic causes of acute paraplegia are well-characterized in the literature, non-traumatic paraplegia cases remain diagnostically challenging because of their broad range of etiologies and limited representation in published studies [3,4]. Among the less common causes, vascular myelopathy accounts for approximately 5-8% of all acute myelopathies and may be overlooked in the differential diagnosis of non-traumatic acute paraplegia [1,4,5].

Leriche syndrome, also known as aortoiliac occlusive disease (AIOD), typically presents with gradually progressive lower limb claudication, diminished femoral pulses, and erectile dysfunction [6]. However, acute presentations of Leriche syndrome have rarely been reported and may manifest as non-traumatic acute paraplegia, which has been reported in approximately 1.8% of cases of aortic occlusion [3,6,7].

In this case report, we describe a rare presentation of non-traumatic acute paraplegia associated with acute Leriche syndrome. This case underscores the importance of considering vascular etiologies in the differential diagnosis of patients presenting to the emergency department (ED) with acute lower limb paralysis, particularly when vascular abnormalities such as absent peripheral pulses and limb coldness are present.

This case was previously presented as an oral presentation at the Malang Integrated Education in Neurology Meeting XIII (MIELUM XIII), held in Malang, Indonesia, on October 25-26, 2025.

## Case presentation

A 58-year-old woman with a history of uncontrolled diabetes presented to the ED with sudden-onset paraplegia. The patient reported that the paraplegia began upon awakening from sleep approximately 14 hours before presentation. Before going to sleep, she had experienced only mild bilateral leg pain, scale 3 out of 10, without any weakness. She denied any recent trauma and reported no history of fever during the preceding two months.

On admission, her vital signs were within normal limits. Neurological examination revealed flaccid paraplegia with muscle strength graded 0/5. Bilateral patellar and ankle reflexes were absent, with bilateral flexor plantar and no pathological reflexes, including a negative Babinski sign. Sensation to pain, light touch, deep touch, and temperature was absent below approximately the L1 level. There was no evidence of sacral sparing, with absent S4-S5 sensation, deep anal pressure, and voluntary anal contraction and tone. A distended bladder was noted, and urinary retention was confirmed following urinary catheterization. Additionally, a focused vascular examination revealed that both lower extremities were cold, with absent bilateral femoral and distal pulses.

**Figure 1:**
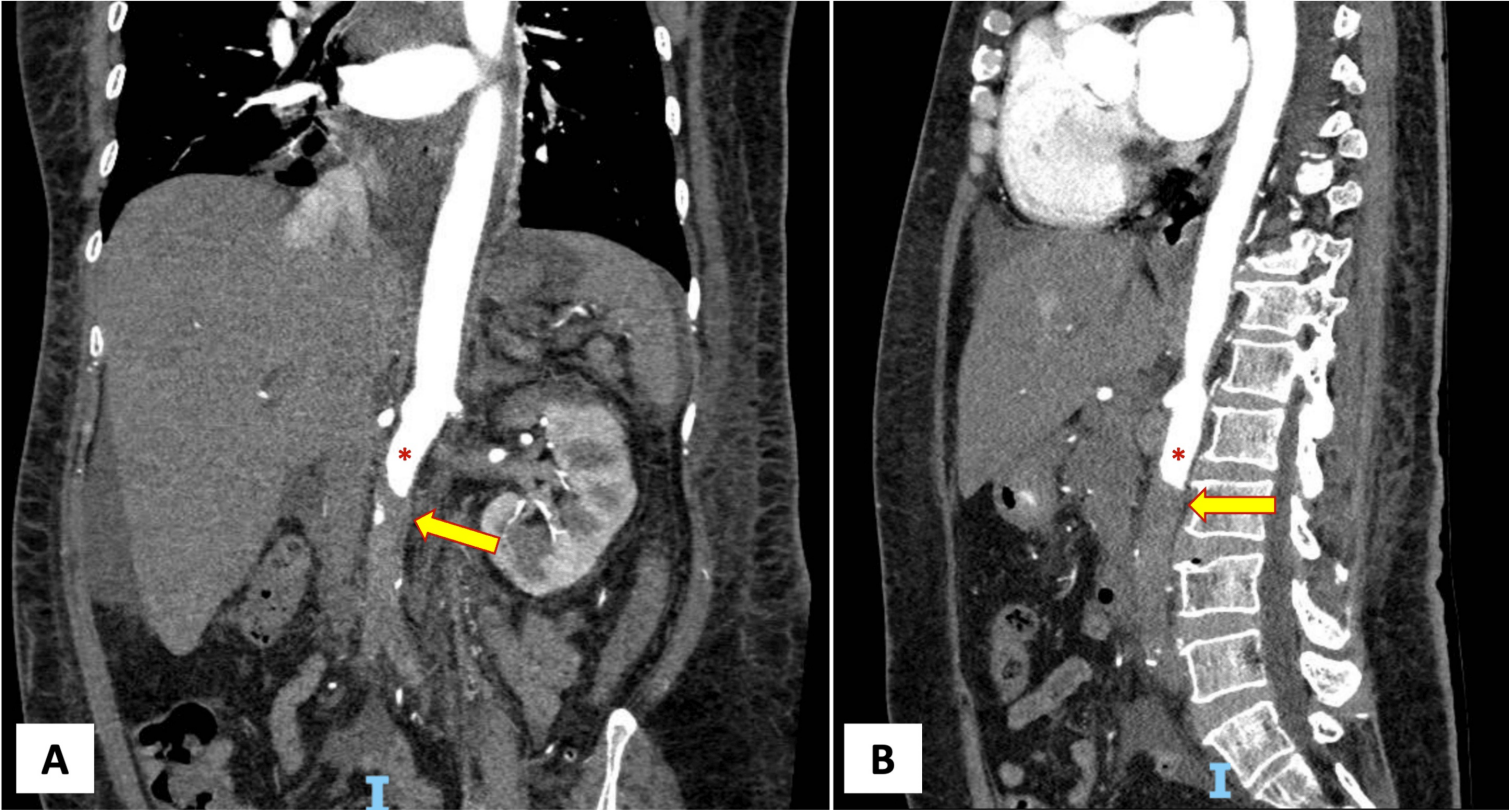
Computed tomography angiography demonstrating acute aortoiliac occlusion Coronal (A) and sagittal (B) views of CT angiography demonstrate contrast opacification of the abdominal aorta proximal to an abrupt cut-off at the L1-L2 level (yellow arrows), consistent with acute occlusion of the abdominal aorta. Red asterisks indicate the contrast-opacified abdominal aorta proximal to the occlusion. The complete CT angiography examination demonstrated associated bilateral common iliac artery occlusion.

An initial clinical diagnosis of non-traumatic acute paraplegia was made. Lumbosacral radiography showed no abnormalities. Laboratory investigations on ED admission revealed a normal complete blood count and liver and kidney function tests. Serum potassium level was also within the normal range (4.29 mmol/L, normal: 3.5-5.1 mmol/L). However, her random blood glucose was elevated at 369 mg/dL (normal: <200 mg/dL), and her D-dimer level was markedly increased (22.76 mg/L; normal: <0.5 mg/L) (Table 1). Given the sudden onset of neurological deficits accompanied by clinical findings highly suggestive of a vascular etiology, a computed tomography (CT) angiography was subsequently performed. Imaging demonstrated a complete occlusion of the abdominal aorta and bilateral common iliac arteries at the L1-L2 level, consistent with acute Leriche syndrome (Figure 1).

**Table 1:** Laboratory findings on admission

Laboratory Parameter	Patient Value	Reference Range
Hemoglobin	13.3 g/dL	11.4 - 15.1
Hematocrit	41.9 %	38.0 - 42.0
White Blood Cell Count	10.33 x 10^3^/µL	4.7 - 11.3
Platelet Count	153 x 10^3^/µL	142 - 424
Prothrombin Time (PT)	13 seconds	9.4 - 11.3
International Normalized Ratio (INR)	1.27	< 1.5
Activated Partial Thromboplastin Time (aPTT)	26.9 seconds	24.6 - 30.6
D-dimer	22.76 mg/L	⪯ 0.5
Aspartate Aminotransferase (AST)	55 U/L	10 - 60
Alanine Aminotransferase (ALT)	35 U/L	10 - 60
Albumin	3.54 g/dL	3.5 - 5.2
Random Blood Glucose	368 mg/dL	< 200
Urea	38.8 mg/dL	16.6 - 48.5
Creatinine	0.69 mg/dL	0.51 - 0.95
Sodium (Na)	135 mmol/L	135 - 145
Potassium (K)	4.29 mmol/L	3.5 - 5.1
Chloride (Cl)	102 mmol/L	98 - 107

Finally, the patient was diagnosed with acute Leriche syndrome presenting with non-traumatic acute paraplegia, with a presumed vascular ischemic mechanism underlying her neurological deficits. Spinal magnetic resonance imaging (MRI) was not performed during the acute evaluation; therefore, spinal cord ischemia could not be radiologically confirmed. The patient was rapidly started on intravenous unfractionated heparin (initial bolus 80 IU/kg followed by continuous infusion at 18 IU/kg/h) with the goal of achieving an activated partial thromboplastin time (aPTT) of 1.5-2.5 times the baseline value. She subsequently underwent successful catheter-directed thrombolysis (CDT) and aortic stent placement.

Her neurological symptoms gradually improved following the intervention; these subsequent improvements following revascularization further supported the clinical diagnosis and association between the acute aortoiliac occlusion and the patient's neurological deficits. Further echocardiographic evaluation demonstrated the presence of a left ventricular (LV) apical thrombus, with LV ejection fraction of 34% and regional wall motion abnormalities involving the basal to mid apical inferior and inferolateral segments. The available echocardiographic report did not document the dimensions or mobility characteristics of the thrombus. The presence of an LV apical thrombus raised the possibility of a cardioembolic contribution to the acute aortoiliac occlusion; however, the available data did not allow definitive differentiation between cardioembolism and in-situ thrombosis. Anticoagulant therapy was continued, and cilostazol was initiated.

After 14 days of hospitalization, she was discharged with muscle strength improved to 3/5 in both legs, and at the three-month follow-up, the patient demonstrated substantial neurological recovery, with restoration of sensory and sacral function, including resolution of urinary retention. However, motor recovery remained incomplete, with muscle strength of 4/5. She could perform some activities independently, but the residual motor weakness resulted in an increased risk of falls and limited her ability to safely perform certain daily activities without assistance. She continued physiotherapy at the time of follow-up.

## Discussion

This case illustrates a diagnostically challenging presentation of non-traumatic acute paraplegia associated with acute aortoiliac occlusion in Leriche syndrome, with a presumed vascular ischemic mechanism. This vascular etiology is not commonly considered in the emergency or neurological settings. The sudden onset of bilateral lower limb weakness with sensory loss and absent reflexes initially mimicked myelopathy, which is more commonly considered in spinal emergencies. However, the absence of distal pulses and limb coldness pointed toward a vascular origin. For emergency physicians and neurologists, this case emphasizes the need for a high index of suspicion for vascular causes of non-traumatic acute paraplegia, where early identification can directly influence prognosis and functional recovery.

Diagnosing non-traumatic acute paraplegia in the emergency setting is challenging, as it involves a wide differential diagnosis and has a broad spectrum of non-spinal pathologies that can mimic spinal cord disease [3,4]. In the emergency setting, a practical approach is to categorize potential etiologies into compressive and non-compressive causes. Compressive myelopathy may result from neoplastic, degenerative, or epidural pathologies, whereas non-compressive etiologies include vascular and inflammatory disorders, as well as myelopathy mimics such as metabolic or electrolyte disturbances, toxic exposures, and paraneoplastic syndromes [1-4].

Among all etiologies of non-traumatic acute paraplegia, compressive myelopathy accounts for the highest incidence (25%), followed by polyneuropathy (20.4%), inflammatory causes (15.9%), metabolic or electrolyte disturbances (9.1%), vascular etiologies (5-8%), and others [4,5]. Given this broad differential diagnosis, the diagnostic evaluation should be guided by the clinical presentation while prioritizing time-critical and potentially reversible causes. Although compressive myelopathy generally requires urgent consideration, an immediate vascular assessment should be performed when vascular red flags are present, such as hyperacute symptom onset, severe limb pain, cold extremities, or diminished or absent femoral or distal pulses. In the absence of such findings, or when the initial vascular assessment is inconclusive, further evaluation should systematically consider compressive myelopathy, followed by non-compressive etiologies, beginning with toxic/metabolic causes, further assessment of vascular causes, and ending with evaluation for infectious or inflammatory processes [1]. In this patient, the cold bilateral lower extremities and absence of femoral and distal pulses strongly suggested a vascular etiology, prompting vascular imaging and subsequent identification of acute aortoiliac occlusion.

Vascular causes account for approximately 5-8% of all acute myelopathies, and among those, spinal cord infarction is often under-recognized [5]. The spinal cord receives its blood supply predominantly from the anterior spinal artery, which arises from segmental arteries fed by the vertebral, intercostal and lumbar arteries. The artery of Adamkiewicz, which most commonly originates between the T8 and L2 spinal levels, is the principal arterial blood supply for perfusing the thoracolumbar spinal cord [5,6,8]. Occlusion of the abdominal aorta and its branches may compromise segmental arterial inflow to the spinal cord (including the artery of Adamkiewicz), potentially resulting in spinal cord ischemia and acute neurological deficits of non-traumatic acute paraplegia [6,9].

Leriche syndrome, also known as AIOD, is classically characterized by the triad of claudication, diminished femoral pulses, and erectile dysfunction in males. However, in rare cases, it may present as an acute presentation with atypical symptoms ranging from erectile dysfunction and paraplegia to sudden multisystem vaso-occlusive catastrophe. Non-traumatic acute paraplegia occurs in only 1.9% of aortic occlusion cases related to Leriche syndrome [7,9]. A hyperacute onset of non-traumatic acute paraplegia (nadir < 4 hours) typically suggests a vascular etiology such as spinal cord ischemia. When vascular etiology is suspected, CT angiography remains the imaging modality of choice in the emergency setting for evaluating aortic and large vessel occlusions [1,10,11].

In our case, the patient had no prior history of claudication and only experienced mild pain prior to sleeping, making the diagnosis particularly challenging. However, the sudden onset of symptoms, complete absence of femoral and distal pulses, coldness in both lower limbs, and elevated D-dimer levels raised clinical suspicion for an acute vascular event. CT angiography subsequently demonstrated complete occlusion of the abdominal aorta and bilateral common iliac arteries at the L1-L2 level, consistent with acute Leriche syndrome.

Management of acute aortic occlusion typically involves prompt systemic anticoagulation, followed by endovascular intervention or open surgical revascularization, depending on the extent and location of the thrombus [6,12,13]. Addressing underlying risk factors are also essential [13]. Our patient was managed with catheter-directed thrombolysis and aortic stent placement after systemic intravenous heparin was initiated. The presence of an LV apical thrombus in echocardiographic findings also raised the possibility of cardioembolic contribution to the acute aortoiliac occlusion, even though the available data did not allow definitive differentiation between cardioembolism and in-situ thrombosis. Long-term anticoagulation therapy was continued, and cilostazol, a phosphodiesterase III inhibitor which has antiplatelet and vasodilatory properties, was added to enhance peripheral perfusion and prevent recurrence [7,9,13].

Although spinal MRI was not performed and spinal cord ischemia could not be radiologically confirmed, the subsequent neurological improvement following revascularization supported the presumed vascular ischemic mechanism underlying the patient's neurological deficits. Acute aortic occlusion associated with Leriche syndrome may therefore present with neurological deficits that mimic spinal cord emergencies and can result in a favorable outcome if detected early and managed appropriately [6,9,14]. In this case, our patient demonstrated significant motor improvement (from 0/5 to 3/5) by the time of discharge and continues to improve during the three-month follow-up, including resolution of sensory and sacral function and urinary retention, with ongoing physiotherapy.

**Figure 2:**
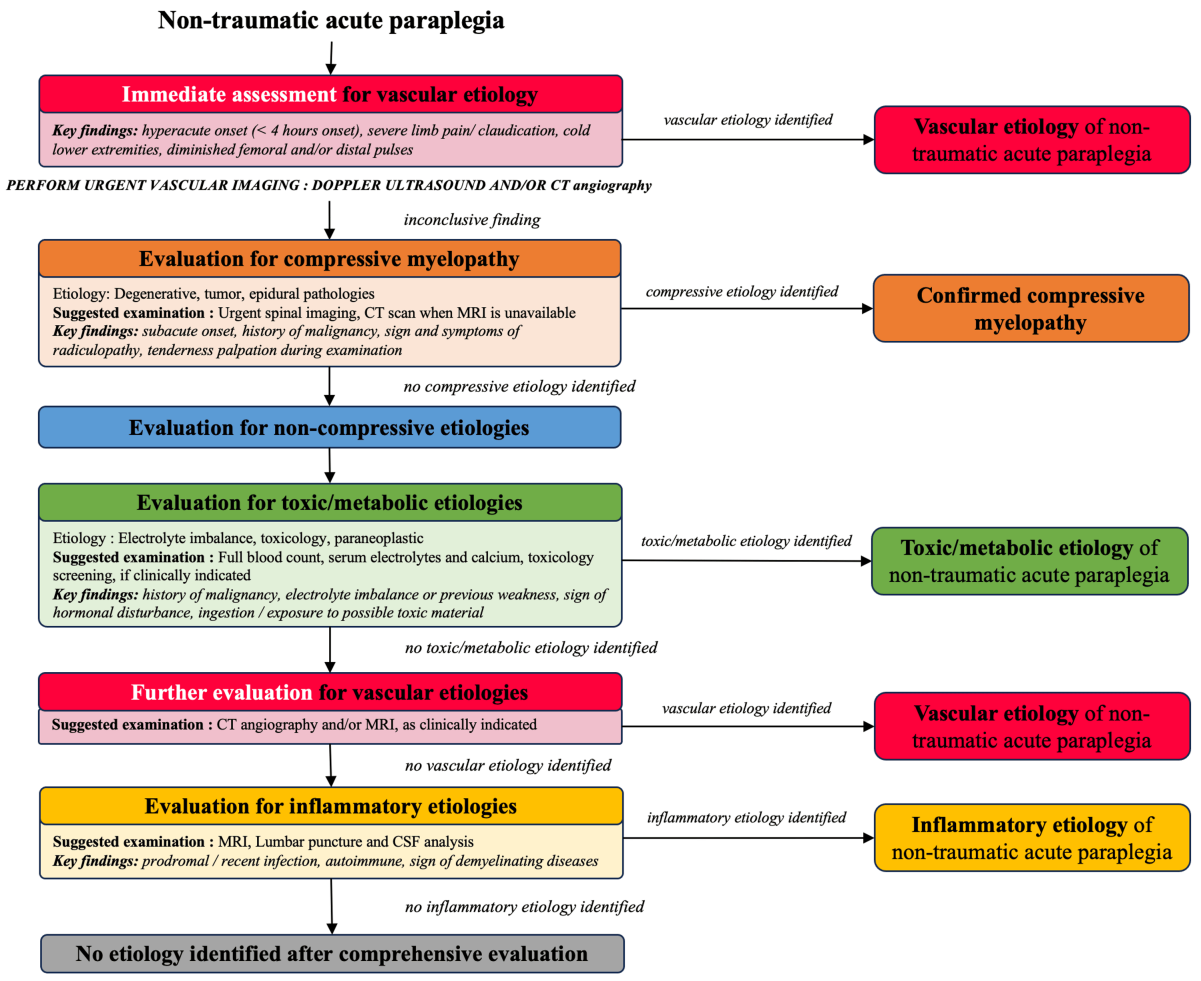
Proposed practical, stepwise diagnostic approach to non-traumatic acute paraplegia in the emergency department The algorithm emphasizes immediate vascular assessment when vascular red flags (cold extremities, absent femoral/distal pulses, hyperacute onset, or severe limb pain) are present before proceeding with the conventional evaluation of compressive and non-compressive etiologies. Image created by the authors using Microsoft PowerPoint (Microsoft Corporation, Redmond, WA, US).

From an emergency medicine perspective, this case emphasizes the importance of maintaining a broad differential diagnosis in patients presenting with non-traumatic acute paraplegia. In resource-limited emergency settings, the diagnostic approach should be rapid, systematic, and cost-effective, with priority given to identifying time-critical and reversible causes. We propose a practical diagnostic framework tailored for physicians in emergency departments that incorporates an immediate focused vascular assessment alongside the initial neurological evaluation (Figure 2). The presence of vascular red flags, including cold lower extremities and diminished or absent femoral or distal pulses, should prompt urgent investigation for an underlying vascular etiology. In the absence of such findings, further evaluation should be guided by clinical suspicion for compressive myelopathy, followed by targeted laboratory testing for toxic/metabolic etiologies, further vascular assessment, and inflammatory workup. This approach may facilitate early recognition of uncommon but potentially reversible vascular causes of acute paraplegia while avoiding unnecessary delays in definitive imaging and treatment [1].

In this patient, the combination of sudden-onset non-traumatic paraplegia, bilateral cold lower extremities, and absent femoral and distal pulses provided critical clues to underlying vascular etiology and prompted CT angiography, which identified acute aortoiliac occlusion. This case demonstrates that acute Leriche syndrome should be considered in patients presenting with non-traumatic acute paraplegia. Applying a structured algorithm enabled early recognition of the vascular etiology, particularly when signs of impaired lower limb perfusion are present and initial clinical, laboratory, and radiological findings were inconclusive for compressive, metabolic, or inflammatory causes. Although this algorithm was developed from a single case, it is intended as a practical emergency department framework rather than a validated clinical decision rule and requires prospective evaluation. Early multidisciplinary collaboration, involving emergency medicine, neurology, cardiology, and vascular surgery, was critical for achieving a positive outcome for this patient.

## Conclusions

A structured, stepwise approach for non-traumatic acute paraplegia in the emergency department is essential for achieving a rapid and accurate diagnosis. Although rare, acute Leriche syndrome can present as acute paraplegia and closely mimic a spinal cord emergency. This case underscores the critical importance of incorporating early vascular assessment into the diagnostic evaluation of non-traumatic acute paraplegia, particularly when signs of impaired lower limb perfusion, such as cold extremities and absent femoral or distal pulses, are present. Early recognition of vascular etiology may facilitate timely revascularization and improve neurological outcomes.
